# Action can amplify motion-induced illusory displacement

**DOI:** 10.3389/fnhum.2014.01058

**Published:** 2015-01-13

**Authors:** Franck Caniard, Heinrich H. Bülthoff, Ian M. Thornton

**Affiliations:** ^1^Max Planck Institute for Biological CyberneticsTübingen, Germany; ^2^Department of Brain and Cognitive Engineering, Korea UniversitySeoul, South Korea; ^3^Department of Cognitive Science, University of MaltaMsida, Malta

**Keywords:** motion-induced position shifts, motion illusions, active vision, local-global motion, perception-action, game psychophysics, mobile devices, closed-loop control

## Abstract

Local motion is known to produce strong illusory displacement in the perceived position of globally static objects. For example, if a dot-cloud or grating drifts to the left within a stationary aperture, the perceived position of the whole aperture will also be shifted to the left. Previously, we used a simple tracking task to demonstrate that active control over the global position of an object did not eliminate this form of illusion. Here, we used a new iPad task to directly compare the magnitude of illusory displacement under active and passive conditions. In the active condition, participants guided a drifting Gabor patch along a virtual slalom course by using the tilt control of an iPad. The task was to position the patch so that it entered each gate at the direct center, and we used the left/right deviations from that point as our dependent measure. In the passive condition, participants watched playback of standardized trajectories along the same course. We systematically varied deviation from midpoint at gate entry, and participants made 2AFC left/right judgments. We fitted cumulative normal functions to individual distributions and extracted the point of subjective equality (PSE) as our dependent measure. To our surprise, the magnitude of displacement was consistently larger under active than under passive conditions. Importantly, control conditions ruled out the possibility that such amplification results from lack of motor control or differences in global trajectories as performance estimates were equivalent in the two conditions in the absence of local motion. Our results suggest that the illusion penetrates multiple levels of the perception-action cycle, indicating that one important direction for the future of perceptual illusions may be to more fully explore their influence during active vision.

## Introduction

Motion-induced position shifts (MIPS) are a class of visual illusion that have long intrigued scientists (e.g., Matin et al., 1976; Freyd and Finke, 1984; Ramachandran and Inada, 1985; Bülthoff et al., 1989; Fröhlich, 1923; De Valois and De Valois, 1991; Nijhawan, 1994; Müsseler and Aschersleben, 1998; Whitney and Cavanagh, 2000, 2002; Thornton, 2002; Müsseler and Kerzel, 2004; see Whitney, 2002; Burr and Thompson, 2011 for reviews). Today, such effects continue to promote important insights into how motion and position interact during object localization, particularly with respect to the level(s) of processing at which such interactions arise (e.g., Arnold et al., 2007; Eagleman and Sejnowski, 2007; Mather and Pavan, 2009; Shapiro et al., 2010; Tse et al., 2011; Kosovicheva et al., 2012; Maus et al., 2013a,b; Li et al., 2014). The current paper is concerned with one specific visual illusion where local motion *within* an object causes a shift in its perceived global position (Ramachandran and Anstis, 1990; De Valois and De Valois, 1991).

**Figure 1:**
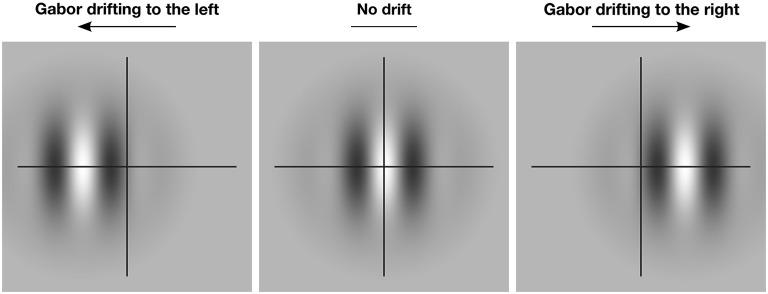
**The effect of motion-induced illusory displacement on perceived global position**. The crosshair indicates the true global position of the Gabor patch. When the patch drifts locally to the left (left panel), the global position of the patch is also shifted to the left. When the patch drifts locally to the right (right panel), the global position of the patch is shifted to the right. In the absence of local drift (center panel) perception of the global position is veridical.

Figure 1 illustrates this basic effect. When the Gabor patch within the aperture is stationary, the perceived global position of the object is veridical. However, if the patch drifts to the left or right within the aperture, the perceived position of the whole aperture is also shifted in the same direction. While the magnitude of this type of illusory position shift is typically quite small—ranging from 2 to 15 min arc for centrally presented targets (De Valois and De Valois, 1991; Tsui et al., 2007; Kerzel et al., 2008)—it is highly robust and has proven particularly useful for exploring the level of visual processing that gives rise to MIPS (e.g., Fu et al., 2004; Arnold et al., 2007; Tsui et al., 2007; Mather and Pavan, 2009; Kosovicheva et al., 2012; Maus et al., 2013a,b).

Recently, we demonstrated that such illusory position shifts continue to affect performance even when participants are given direct, active control of the object’s global position (Caniard et al., 2011). That is, action does not eliminate this form of illusion (see also, Yamagishi et al., 2001). In our previous study we created a simple 2D tracking game where participants used a joystick to guide a Gabor patch along a winding path. Our dependent measure was deviation from the path, sampled at 40 Hz. When the Gabor patch was stationary, participants could perform this task very accurately. When we introduced local drift to either the left or right, however, there were consistent shifts. Specifically, drift to the left caused participants to make a compensatory correction and to position the patch to the right of the line, drift to the right caused the opposite correction. The effect was highly consistent across participants, of a magnitude similar to that reported for perceptual tasks (between 8–15 min arc, depending on drift speed) and did not vary with time-on-task.

The goal of the current paper was to directly compare the magnitude of the illusion that persists under active control to that obtained in more typical scenarios where the participant is a passive observer. Although the manual tracking used in Caniard et al. (2011) provided a simple and intuitive way to measure action-related shifts, the continuous nature of the path was not amenable to creating a perceptual control task. Thus, although we were able to demonstrate that the illusion persisted in an active scenario, we could only calibrate the size of the illusion with reference to previous perceptual studies. Here, our aim was to measure both active and passive forms of the illusion using the same display and the same participants.

**Figure 2:**
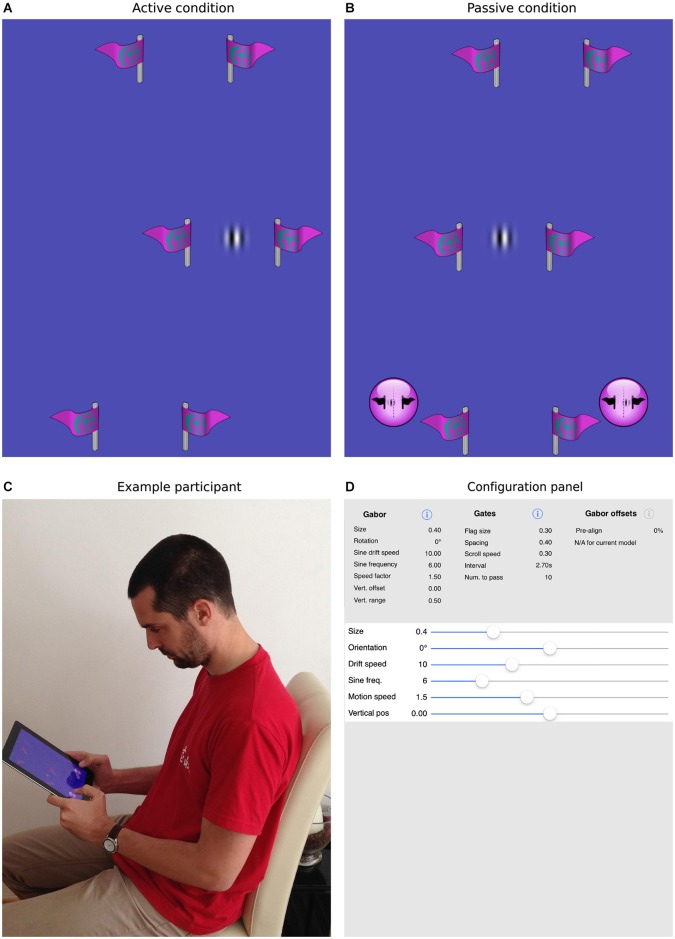
**The current task and mobile app**. Panels **(A,B)** show screenshots of the task, where the patch is either actively guided through the gates **(A)** or is driven through the gates under program control **(B)**. In the former case, the participant is required to steer the patch directly through the center of the gates. In the latter case, the participant uses the virtual buttons at the bottom of the screen to indicate whether the patch passed to the left or right of center. Panel **(C)** shows a participant interacting with an iPad, here performing the passive task. Panel **(D)** illustrates how all parameters within the app, for example, the size, orientation, spatial/temporal frequencies of the target patch, the number, size, spacing and frequency of the gates, can be selected and parametrically varied to create a wide range of experimental situations.

The task we designed to achieve this goal is illustrated in Figure 2. As in simple video games, participants were presented with a top-down view of a scene in which a target object appeared to move continuously through the environment. In fact, the vertical position of the target Gabor patch was fixed at the center of the screen and forward motion was implied by scrolling a series of slalom-like gates (two symmetrical flags made of a vertical stick and a flag pointing outwards) at a constant rate from the top to the bottom of the display. In separate blocks, the sine-wave component of the Gabor patch was either stationary or drifted to the left or right.

During our active task, participants could control the horizontal position of the patch by physically tilting the device to the left or right. We directly measured deviation from midline each time the patch entered a gate. In the passive task, the same scene was presented, but participants had no control over the behavior of the patch. Rather, they watched automatically generated trajectories in which the alignment of the patch with the gate midline was parametrically varied. Two virtual buttons were used to make left/right judgments and we measured the influence of patch drift by fitting cumulative normal curves to the distributions of responses and establishing the point of subjective equality (PSE). The same 12 participants completed both tasks in a counterbalanced order allowing us to directly compare illusory shifts with and without the influence of action.

## Methods

### Participants

Twelve volunteers from the Swansea University community, aged from 18 to 47 years (8 male; *M* = 24.8 years, SD = 9.5 years) participated in this experiment. All reported normal or corrected to normal vision, were right handed and gave written, informed consent. All aspects of the experiment were reviewed and approved by the departmental ethics committee at the University of Swansea and thus conformed to the ethical guidelines set out by the Declaration of Helsinki for testing human participants.

### Equipment

The experiment was conducted using an iPad 2 (Model number A1395) with a screen diagonal length of 9.7 inch (197 × 148 mm), and a resolution of 1024 × 768 pixels. Graphics were rendered using the highly optimized OpenGL ES graphical library to provide a constant frame rate of 60 Hz. The device was held freely in both conditions, and head/eye position was not monitored or constrained (Figure 2C). Thus, we could only estimate the average viewing distance to be roughly 50 cm. Although we report stimuli and results in terms of visual angle—to follow the previous literature—it should be noted that here these values are only an approximation. During the description of the stimuli, we have also provided size information in terms of mm and device specific pixels. For the sake of brevity, only degrees or arcminutes of visual angle are reported in the results section.

### Custom iPad application

A custom iOS app was written to present the task and collect data. The app was implemented in Objective C using Xcode and OpenGL ES libraries. All aspects of the experiment, including the size, orientation, spatial/temporal frequencies of the target patch, the number, size, spacing and frequency of the gates and range and number of repetitions of the passive offsets were designed to be selectable via standard iOS interfaces (see Figure 2D). Parameter files, used to control the experimental sessions, were also designed to be easily imported and exported in order to share settings. Both the app itself and the source code are freely available from the MPI for other researchers to replicate the current design or to experiment with new settings. Please contact the corresponding authors for more information.

### Stimuli

#### Target patch

The target object was a low-frequency Gabor patch. That is, a vertical sine wave grating that was windowed by a Gaussian envelope to produce a circular patch with undefined edges. The spatial frequency of the Gabor was constant throughout all conditions at 1.9 cycles/° and had a visible extent of approximately 1.6° (1.4 cm, 74 pixels) in diameter. The grating could be either static, or drifting from left to right or right to left. The temporal frequency of this drift was 1.6 cycle/s, and was the same across all conditions.

#### Gates

The size and scrolling speed of the gates as well as the time between two consecutive gates was the same for all conditions. The width of each gate was set at 4.2° (3.7 cm, 192 pixels), providing ample space for the target patch to pass unobstructed. The height of each gate was set at 2.5° (2.2 cm, 115 pixels). The gates scrolled at a constant speed of 2.07 cm/s, with an interval of 4 s between consecutive gates. These parameters were determined in pilot testing so that there would be no difficulty in moving from one gate to the next. The horizontal position of each gate was set randomly just before it appeared on the screen within a range that guaranteed that both flags were visible for all gates.

### Response and patch control

During the active task, the goal was to adjust the position of the target patch so that it passed exactly through the center of the approaching gate. The position of the patch was adjusted by using the iPad’s built-in tilt control. Only the horizontal position of the patch could be adjusted. Physically tilting the device to the left or right caused the patch to move in the same direction. Larger tilt angles increased the speed of patch movement. If the patch reached the edge of the screen or if the device was held flat, relative to the participant, the patch remained stationary. The vertical position of the patch was fixed in the middle of the display. We adopted this simplification to reduce between-participant variability in global patch positioning and to make it more straightforward to approximate average patch behavior in the passive condition.

During the passive task, participants continued to hold the iPad and the same scene was presented, with the addition of two non-intrusive virtual buttons, one at the bottom left and one at the bottom right of the screen. The participants had no control over the behavior of the patch. Rather, the exit trajectory from each gate was calculated automatically to follow a smooth path that stabilized in front of the next, approaching gate. The parameters for this linking trajectory were established prior to the main experiment and were based on the average behavior of a number of pilot participants. The endpoints of the linking trajectories were parametrically varied to adjust the horizontal alignment of the patch with the center of the approaching gate. In a random order, the patch could be physically aligned, or shifted to the left or right of the gate center. During the transit through the gate, this alignment remained constant. The participant’s task was to report the direction of perceived offset by pressing either the right or the left button once the patch had entered the gate. For a response to be recorded, button presses had to occur while the patch was physically overlapping with a gate.

### Task and design

Each participant completed both active and passive tasks in an order that was counterbalanced, so that an equal number started in both conditions. Each task began and finished with a control condition in which the patch did not drift. The pre-test condition helped familiarize participants with the display and response demands of the main condition and provided baseline information on how well the task could be performed in the absence of the illusion. The post-test condition measured effects of learning or fatigue on overall task performance, again in the absence of local drift. Data on both gate and patch position was collected after each frame, allowing us to reconstruct the entire trajectory through all sessions.

### Active task

The pre- and post-test phases involved guiding the patch through a total of 30 gates. If a gate was not entered or there was a collision between the patch and a gate, then data was not recorded and the gate was replaced later in the experimental design. The patch did not drift during these trials. The task was to try and position the patch to exactly pass through the center of each gate. The pre- and post-test phases lasted approximately 2 min each.

In the main experimental phase, participants guided the patch through a total of 160 gates. The patch drifted to the right or left at a constant speed, alternating direction every 20 gates. Initial drift direction was randomized, and a short break was provided after 80 gates had been successfully completed. All other aspects of the task were the same as for pre- and post-tests. This phase of the experiment lasted approximately 15 min.

### Passive task

The pre- and post-test phases involved observing the patch pass through a total of 54 gates. The alignment of the patch with the gate center was parametrically varied, and each of seven offset values (−18, −12, −6, 0, 6, 12, 18 arcmin), appeared eight times. The order in which the offset values appeared was determined randomly for each participant. The task was to indicate whether the patch passed to the left or right of the gate center using the appropriate virtual button. As for the active condition, the patch did not drift during these trials. If a response occurred before a gate or once a gate had been traversed, the response was ignored and the gate replaced later in the experimental design. The pre- and post-test phases lasted approximately 4 min each.

In the main experimental phase, participants observed the patch passing through a total of 168 gates. The patch drifted to the right or left at a constant speed, alternating direction after every 21 gates. All offsets were presented within this block of 21 gates, with each offset repeating three times in a separately randomized order. Initial drift direction was randomized, and a short break was provided after 84 gates. All other aspects of the task were the same as for pre- and post-tests. This phase of the experiment lasted approximately 15 min.

As for the active task, data on both gate and patch/offset position was collected after each frame, which allowed us to reconstruct the entire trajectory through all passive sessions.

### Procedure

Participants were run in individual sessions, lasting approximately 45 min. Written instructions were provided to give an overview of the experimental demands and written informed consent was obtained before testing commenced. Participants were recruited through opportunity sampling and the sessions took place in a variety of locations in and around Swansea University campus, taking advantage of the mobile nature of our experimental set-up. Where possible, a quiet location away from direct overhead lighting was used.

At the start of both the active and passive tasks, a brief, hands-on demonstration was provided in which the participant performed under close supervision. During this familiarization phase, the patch did not drift, and feedback was provided both by the experimenter and in the form of on-screen visual cues. The experimenter gave general feedback if it appeared the participant did not understand the task or was not performing as instructed. The on-screen feedback only related to gates that were missed during the active task or for which a response was not made during the passive task. During the active demonstration, an additional icon—an upside-down flag—was added to each gate that was successfully traversed. The absence of this flag thus served as feedback. During passive trials, the same icon was added to gates that did not receive a response.

When the participant appeared comfortable with the task (usually in less than 20 gates) the relevant pre-test session was conducted. This was followed by the main experimental phase. Before this phase, participants were shown that the patch would drift to the left or right but were instructed to perform the task as for the pre-test. Following the final post-test phase, during which the patch did not drift, participants were provided with a written debriefing sheet and were given the opportunity to ask questions about the task and the research project in general. Self-paced breaks were included between each phase of the experiment.

#### Analysis

The order of the two tasks were counterbalanced across participants, with six completing the active task first, and six completing the passive task first. We also ran initial analysis with order as a between subjects factor. However, as there were no main effects or interactions involving this factor, we have omitted order from the main analysis reported below.

Consistent with our previous paper (Caniard et al., 2011), estimates of illusion strength in the active task were calculated directly from the manual adjustments that participants made to control the patch as it passed through each gate. All negative values relate to positions to the left of gate center, positive values to the right of gate center.

The dependent measure for the active task was the distance of the patch from the gate center during gate entry, always reported in terms of arcmin. To compute this measure, we averaged the spatial offset from all frames during the first half of each gate. During pilot testing it became clear that during the latter part of each gate participants were already beginning to move the patch to the left or right in preparation for the approach of the next gate. As the initial portion of the gate was relatively stable, it provided a more conservative and less noisy estimate of patch/gate alignment.

The dependent measure for the passive task was the PSE for each condition, obtained by fitting cumulative normal functions to the individual left/right response distributions. We computed goodness of fit for all curves using the deviance measure D and compared these to distributions of D’ generated by a bootstrap method using the parameters of the fitted curve (*N* = 2000; Wichmann and Hill, 2001). All measures of D were within the 95% confidence limits of D’, indicating acceptable goodness of fit. A similar bootstrap technique was used to estimate the standard errors associated with the PSE estimates (*N* = 2000; Prins and Kingdom, 2009).

As it was possible that the participant missed gates in the active case, or failed to give a response in the passive case, sessions were not limited in time or in number of gates, and new gates kept appearing until the software collected the required amount of data for each condition. In reality, the scrolling speed and time interval between gates was set so that participants had little difficulty performing the tasks, and invalid gates or missing responses were rare, occurring on less than 2% of attempted gates in any condition.

One sampled *t*-tests were used to determine if the group means within each condition varied consistently from the centerline of the gates. Paired *t*-tests were used to make simple comparisons between group means. Where appropriate, Bonferroni corrections were applied for multiple comparisons. To explore the pattern of data across condition and time, we used repeated measures Analysis of Variance (ANOVA), applying Greenhouse-Geisser correction to adjust for possible violations of sphericity. Details of the models applied are provided in the text below.

## Results

### Active task

**Figure 3:**
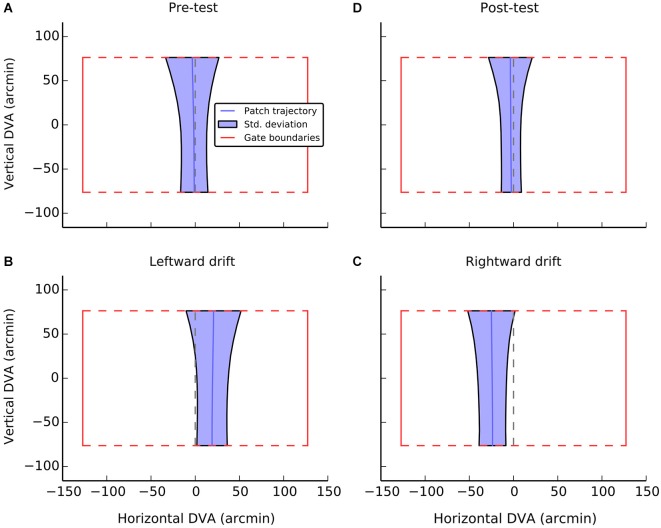
**A visualization of average performance during the active task as a function of experimental condition**. The red box in each panel shows the gate boundaries. The true center of the gate is illustrated by the dashed red line. Performance in guiding the patch, averaged across gate and participants is illustrated with the solid blue line. The shaded blue region indicates the standard deviation of errors at each point during passage through the gate. The broadening of the regions at the top of the gate can be accounted for by preparatory movements determined by the position of the next gate. Data were only analyzed from the first half of each gate passage. **Panels A** and **D** show performance during control conditions, when the patch had no local drift. **Panels B** and **C** show performance during leftward and rightward drift, respectively.

Average performance, collapsed across participant and gate, is visualized in Figure 3. Each panel indicates the dimension of an idealized gate and the average trajectory that was taken through that gate in a given experimental condition. During the pre-test phase (Figure 3A), when there was no local motion, there was essentially no deviation (*M* = −1.3, SE = 1.3) and participants were able to guide the patch through the center of the gate. A one-sample *t*-test confirmed that trajectories did not significantly differ from center (i.e., zero), *t*_(11)_ = −1.0, n.s.

**Figure 4:**
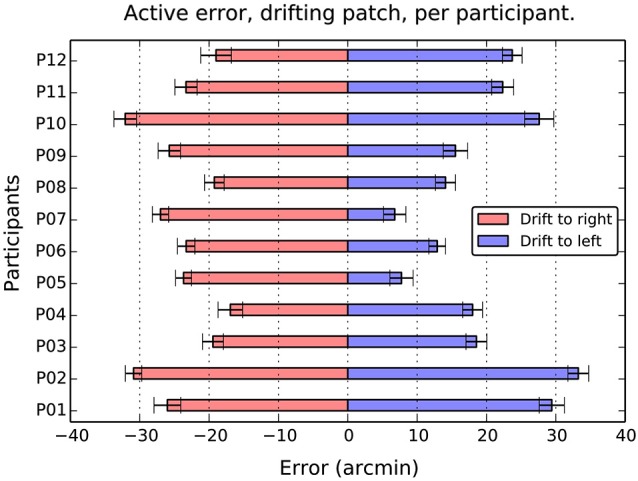
**Average active error as a function of participant and drift direction**. Although there are considerable individual differences in the balance between left and right errors, the overall effect of direction is totally consistent. Drift to the right causes a compensatory shift to the left, and drift to the left, a shift to the left in all 12 participants. Error bars show 1 standard error of the mean.

When the patch drifted to the left (Figure 3B), participants compensated by consistently positioning the patch to the right of center (*M* = 19.2, SE = 2.4, *t*_(11)_ = 7.9, *p* < 0.001). When the patch drifted to the right (Figure 3C), the opposite shift was observed (*M* = −23.9, SE = 4.8, *t*_(11)_ = −17.4, *p* < 0.001). As can be seen in Figure 4, this reversal as a function of drift direction was extremely consistent, occurring for all 12 participants.

To compare the magnitude of shifts, and to explore for effects of learning, we conducted a 2 (Direction: left or right drift) × 4 (Block) repeated measures ANOVA on absolute shift values. There was a marginally significant effect of Direction, *F*_(1,11)_ = 4.2, MSE = 127.3, *p* = 0.06, $\eta_{p}^{2}$ = 0.28, reflecting the slightly larger corrective positioning during rightward drift noted above. There was no main effect of Block, *F*_(3,33)_ = 0.01, MSE = 30.5, *p* = 0.96, $\eta_{p}^{2}$ = 0.01, and no Direction × Block interaction, *F*_(3,33)_ = 0.96, MSE = 29.6, *p* = 0.42, $\eta_{p}^{2}$ = 0.08.

During post-test, the magnitude of shifts returned to the level of pre-test, although there was a small but consistent deviation to the left, that was significantly greater than zero (*M* = −2.9, SE = 1.2, *t*_(11)_ = −2.4, *p* < 0.05). A direct comparison between the pre- and post-test levels, however, indicated that there was no significant difference, *t*_(11)_ = 1.2, n.s. Overall, it seems there is a slight bias to position the patch to the left of center in the absence of local motion. This tendency may also have contributed to the slightly larger shifts observed when the patch was drifting to the right.

### Passive task

**Figure 5:**
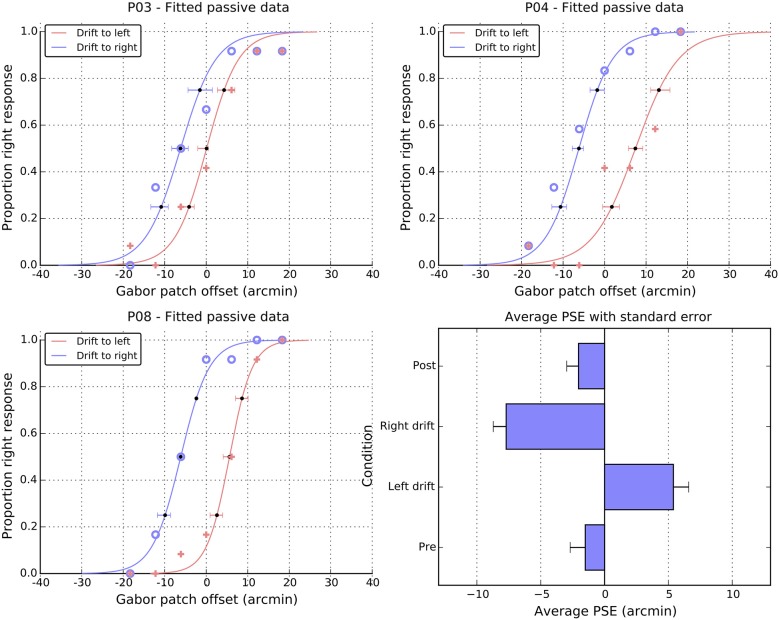
**Individual and average performance in the passive task**. Panels P03, P04, and P08, show the proportion of right responses made by these participants as a function of drift direction and physical offset. Blue open symbols show responses when the patch drift was to the right and pink cross symbols when the patch drift was to the left. The solid lines are cumulative Gaussian fits, and the error bars are 95% confidence intervals, estimated via bootstrapping. The point of subjective equality (PSE) extracted from the fits of individual participants are summarized in the lower right panel as a function of experimental condition (see text for details). Note the change of scale in the panel relative to the individual fits and Figure 2.

Performance in the passive task is summarized in Figure 5. Panels A–C show data from three representative individuals and plot the proportion of “right” responses as a function of the physical offset of the patch. It is clear that participants could differentiate between the physical offsets and more particularly, that greater physical shifts to the right were required when the patch was locally drifting to the left. That is, in all three panels, the solid crosses (left drift) are to the right of the open circles (right drift). To quantify these patterns of shift, we fitted cumulative normal distributions (curves in the individual panels) to the data of each participant and extracted the PSE. Figure 5, panel D shows a summary of the PSE values for all 12 participants, and also includes values for the two static control conditions, which were obtained using the same method.

For both pre- and post-test conditions, the perceived midpoint was very close to veridical. However, while the pre-test value did not differ from zero (*M* = −1.5, SE = 1.2, *t*_(11)_ = 1.2, n.s.), the post-test value was marginally shifted to the left of center (*M* = −2.1, SE = 1.0, *t*_(11)_ = 2.1, *p* = 0.06). Note that both the magnitude and the direction of these baseline shifts are consistent with those observed in the active condition. Here again, in the absence of motion, it appears the patch was perceived slightly to the right of center, leading to a leftwards shift in the position of the curve and PSE. As with the active data, a direct comparison of the magnitudes of pre- and post-test shifts showed that they were not significantly different from each other, *t*_(11)_ = 0.3, n.s.

As noted above, drift to the left meant that participants required more physical shift to the right to perceive the patch as centered, causing a significant shift in the PSE to the right (*M* = 5.4, SE = 1.3), *t*_(11)_ = 4.3, *p* < 0.01. Drift to the right caused the corresponding shift of the PSEs to the left (*M* = −7.7, SE = 1.1), *t*_(11)_ = 7.3 *p* < 0.001. A paired *t*-test on these signed shifts confirmed they were significantly different from each other, *t*_(11)_ = 7.8 *p* < 0.001. However, examining the absolute values suggests that the magnitude of these left and right shifts were not consistently different, *t*_(11)_ = 1.4, n.s.

### Comparing active and passive tasks

**Figure 6:**
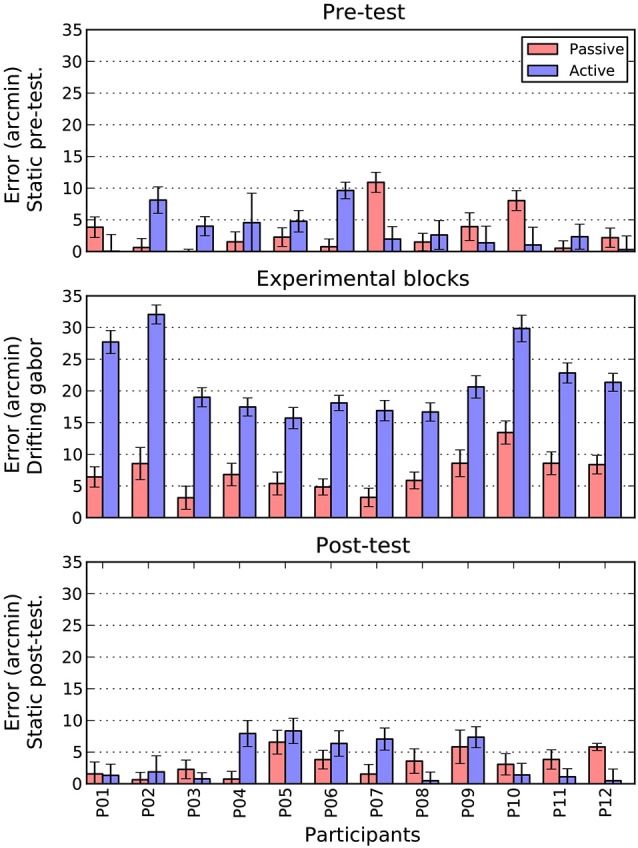
**Comparison of active and passive absolute errors as a function of experimental phase and participant**. Error bars show 1 standard error of the mean, estimated directly (active bars) or via boostrapping (passive bars). See text for details.

The main goal of this paper was to directly compare the magnitude of shifts obtained under active and passive conditions. The results of this comparison can be seen in Figure 6. Here, for both conditions, we collapsed across direction by taking the mean absolute shift as our dependent measure. The three panels show the pre-test, drifting and post-test phases of the experiment, respectively.

There are two patterns that are immediately clear for Figure 6. First, during the pre- and post-test phases, there is no difference in the magnitude of shifts between the active and passive conditions. To confirm this pattern, we conducted a 2 (Task: Active/Passive) × 2 (Phase: Pre-test/post-test) repeated measures ANOVA. There were no significant main effects and no interaction.

The second point to note is that when the patch is drifting, the size of the active shift (*M* = 21.5, SE = 1.6) is more than three times larger than the equivalent passive shift (*M* = 6.9, SE = 0.8). A paired *t*-test confirmed that this difference was significant, *t*_(11)_ = 12.2 *p* < 0.001. To examine whether this increase in magnitude was also accompanied by an increase in variability, we directly compared the standard error estimates obtained in the two conditions. As can be seen in the error bars of Figure 6, the active (*M* = 1.6, SE = 0.1) and passive (*M* = 1.7, SE = 0.1) drifting conditions gave rise to very similar estimates, *t*_(11)_ = 1.5 n.s. However, as these measures were derived very differently in the two conditions (i.e., direct measurement for active and bootstrapping for passive conditions) we also examined the change in variability within each condition between the static baseline trials (pre and post) and drifting trials. Interestingly, while variability increased slightly from static to drifting trials in the passive condition (*M* = 0.2, SE = 0.1), it actually decreased in the active condition (*M* = −0.5, SE = 0.1), *t*_(11)_ = 3.4 *p* < 0.01. Thus, it appears that increased variability does not contribute to the overall increase in shift.

**Figure 7:**
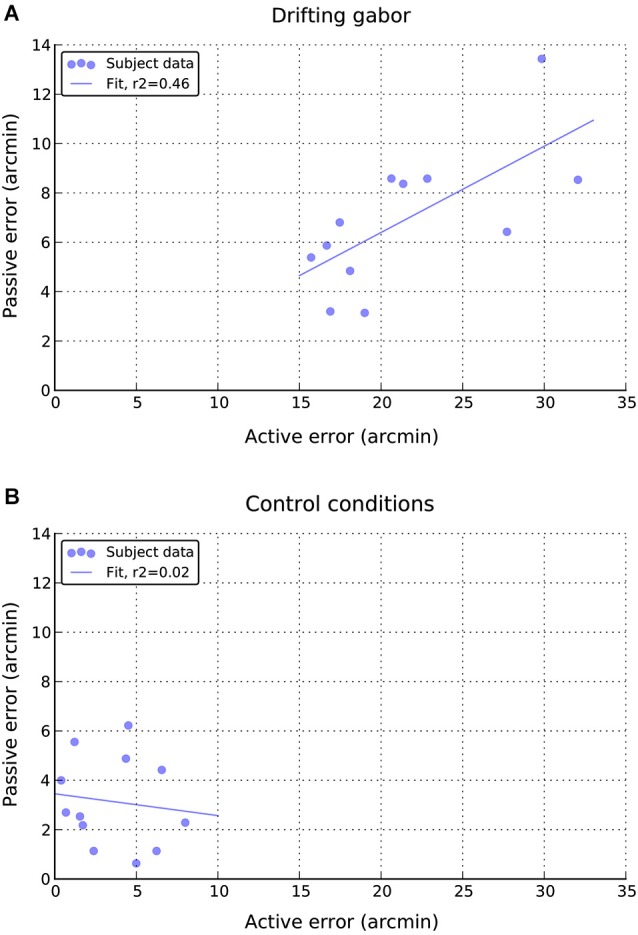
**Relationship between individual active and passive errors during (A) drifting phase and (B) static control phases of the experiment**.

Finally, as can be seen in Figure 7A, there appears to be a moderate positive relationship between performance in the active and passive drifting conditions, such that those participants with larger active shifts also had larger passive shifts, *r*_(10)_ = 0.68, *p* < 0.05. No such relationship is apparent in the static control conditions (Figure 7B), *r*_(10)_ = 0.14, n.s.

## Discussion

There were three main findings in the current study. First, when participants passively judged the alignment of drifting targets as they passed through the gates there was a clear shift in the perceived position, consistent with previous studies using this illusion (e.g., De Valois and De Valois, 1991). Second, as in our previous study (Caniard et al., 2011), we showed that providing direct, active control over the global position of the target object did not eliminate this illusory shift. Rather, participants consistently chose a path through the gates that was shifted in the direction opposite to the local drift. Third, for all participants, the magnitude of the perceived shift was considerably larger in the active compared to the passive task.

Our first finding provides yet another example of the robust nature of this illusion. Although the size of the perceived shift during the passive task was relatively small, even for a centrally viewed target (De Valois and De Valois, 1991; Tsui et al., 2007; Kerzel et al., 2008), the influence of motion on position was extremely consistent, showing the same pattern in all 12 participants. In the absence of motion, there was a slight tendency to perceive the patch to the right of center, a tendency that was more apparent during the post-test phase. This general alignment bias may also have contributed to the left/right asymmetry apparent in the drifting conditions of both passive and active experimental phases. We have no immediate explanation for this finding, but it may be interesting to explore whether handedness plays a role. In the current study, all of our participants were right handed. Examination of the accelerometer data from the iPad might also shed light on whether there was a tendency to tilt the device slightly during the passive task to explore whether subtle differences in orientation may also have contributed.

The adjustment error seen during the active task was of the same order of magnitude as that measured in our previous study (Caniard et al., 2011). Indeed, the current paradigm appears to give rise to slightly larger shifts. However, as we noted in the methods section, viewing distance was not reliably fixed, so some caution is needed when drawing conclusion based on a direct comparison of magnitude. We can be less cautious with respect to another aspect of the data in the two studies. In both sets of data, there was no indication that either the pattern or magnitude of corrective shifts were affected by time-on-task. If participants had access to any cues to indicate a mismatch between the true center and their guidance of the patch, then we might have expected some form of re-calibration (e.g., Welch, 1978; Cunningham et al., 2001; Miall and Jackson, 2006; Fajen, 2007). For example, had the more accurate estimates of patch alignment measured during passive performance been independently available during the active task, these could have been used to improve performance. The lack of re-calibration suggests either that such independent cues were not available or that the process of controlling the patch actually modified the ability to accurately perceive the true center (i.e., amplified the perceptual illusion).

Our third finding—larger shifts in action vs. perception—replicates the pattern of results reported by Yamagishi et al. (2001). In their study, passive judgments of Gabor position were made using a visible ruler and active judgments consisted of ballistic pointing movements. Here, we used very different measures of both active and passive performance, but find a similar relationship between the two types of response. Although Kerzel and Gegenfurtner (2005) found that the nature of the passive probe could have a large influence on whether action yielded bigger, smaller or equal shifts, the current study would appear to add at least one more scenario in which action can amplify motion-induced illusory displacement. More generally, we should note that the influence of action on MIPs does seem to vary across different types of effect. For example, with representational momentum (Freyd and Finke, 1984), action has been consistently shown to produce larger effects than purely perceptual judgments (Kerzel, 2003; Ashida, 2004). However, both larger (Scocchia et al., 2009) and smaller (Ichikawa and Masakura, 2010) illusory effects have been reported when action is coupled with the flash-lag effect (Nijhawan, 1994; Nijhawan and Kirschfeld, 2003).

### Evidence for “two systems”?

Yamagishi et al. (2001) interpreted their findings as further evidence for a dissociation between visual perception and visually guided action (Goodale and Milner, 1992; Milner and Goodale, 1995). Clearly, the current findings could also be interpreted in this way. However, the application of this dichotomy in the context of visual illusions continues to be highly controversial (Bruno, 2001; Carey, 2001; Franz, 2001; Franz et al., 2001, 2003; Smeets and Brenner, 2001; Glover, 2004; Franz and Gegenfurtner, 2008; Cardoso-Leite and Gorea, 2010). More importantly, there are several reasons to believe this is not the most useful framework within which to interpret the current results.

To begin with, MIPS, as a class of motion-based effects, are almost certainly not the product of high-level ventral processing, one of the important prerequisites for exploring two-systems dissociations in the context of illusions (Milner and Dyde, 2003; Milner and Goodale, 2008). On the contrary, there is considerable evidence that MIPS rely extensively on dorsal-stream activity. That is, it seems likely that the locus of such effects are either extremely early, at the stage of motion energy extraction (i.e., V1, e.g., Bülthoff et al., 1989; Jancke et al., 1999; Fu et al., 2004)—a stage that would be shared by both ventral and dorsal streams—or during motion integration in extra-striate regions. In particular, recent TMS (e.g., McGraw et al., 2004; Maus et al., 2013b), fMRI (e.g., Maus et al., 2013a) and behavioral data (Mather and Pavan, 2009) all point to the involvement of human area MT+.

Similarly, the active control task used in the current experiment would appear to fall short of the criteria laid out by Milner and Goodale (2008) for selecting pure dorsal-stream tasks. The authors note that “Only highly practiced actions with the right hand operating in real time and directed at visible targets presented in the context of high-level illusions are likely to escape the intrusion of ventral stream perceptual control” (p 780). As “high-level” in this context refers explicitly to ventral-stream illusions and as our task involves bi-manual control in a novel task, there are clearly several reasons to doubt that performance would be immune to perceptual cross-talk.

Our goal here is not to debate the general utility of the two-systems approach when comparing action and perception (for an alternative “modular” approach see Glasser and Tadin, 2014) but simply to point out that MIPS, particularly with the current experimental design, might be very bad candidates with which to explore such issues. We also note that in addition to the possible theoretical overlap between the two systems mentioned above, our finding of a relatively strong correlation between performance on the two tasks would be another reason not to pursue arguments based solely on separable systems. Regardless of whether the pattern of results is interpreted within the standard perception-action dichotomy or is viewed simply as a consequences of modularity—multiple components of a complex task drawing on non-overlapping resources (Glasser and Tadin, 2014)—the important question to consider is *why* the illusion seems to have a larger effect on the active task than the passive task.

### Methodological explanations

Importantly, our pre- and post-test conditions would appear to rule out several methodological differences unrelated to the illusion. For example, increased shifts could have arisen due to problems in manually guiding the patch or to differences in the precise trajectories used in the two conditions. The fact that pre- and post-test performance was essentially identical, suggests that the source of the amplification must relate to the illusion itself. That is, we only see a difference between the tasks in the presence of local motion.

Although we worked hard to ensure that the displays themselves were as similar as possible between the active and passive conditions, there remained at least two important differences in viewing conditions. First, as participants were required to physically tilt the iPad in order to direct the patch in the active condition, the visual parameters of the display will also have changed. For example, tilting the iPad would induce changes in the visible size and contrast of the Gabor patch, factors that are known to affect the perception of position in the context of MIPS (Arnold et al., 2007; Tsui et al., 2007). Ideally, our passive condition would have reproduced the physical tilt of the device along with simulated patch trajectories. This could be achieved, for example, by using recorded tilt information to drive a robotic arm. An alternative approach would be to eliminate tilt from the active condition by using the virtual buttons, rather than device orientation to guide the patch.

In the current experiment, participants continued to hold the device during passive trials. Although we did not record orientation information for these trials, we assume that device tilt would have been minimal. During active trials, the iPad was mostly held relatively flat (*M* = 0.03°, SE = 0.01; mode = 0.0°), as device tilt was only used to change direction. As can be seen in Figure 3, on approach to a gate, the patch was typically following a straight path when tilt would have been near zero. Nevertheless, the variability in tilt angle was quite high (*M*_Stdev_ = 9.4°, SE = 0.1) and maximum orientation changes of approximately 20° did occur during active trials for all participants (*M*_Max_ = 21.1°, SE = 0.3). It thus remains a possibility that accompanying visual changes contribute to the increase in shift relative to the passive condition.

The second issue with regards to viewing conditions relates to how observers were sampling the stimuli. That is, it remains possible that gaze direction or the allocation of attention varied consistently between the two conditions. For example, if gaze position were shifted towards one or other of the gates in the active condition this might have lead to a corresponding “steering shift” in the same direction (e.g., Readinger et al., 2002). Similarly as the patch was under observer control during active trials, it may have received more fixations than in the passive condition, where only the gate entry was task relevant. The current form of motion-induced illusion is known to scale with eccentricity, increasing by 1–2 arcmin/° (De Valois and De Valois, 1991; Fu et al., 2004; Chung et al., 2007). If the patch was being viewed more peripherally in one condition, this could then lead to a consistent increase in shift. Of course, the direction of the difference we observed, would require that more fixations were made on the patch during the passive condition, which seems counter-intuitive. However, to further explore exactly where observers look during the two conditions we are currently using the app in conjunction with a mobile eye-tracking system. We may also explore the role of attention by introducing additional “probe” events—such as briefly dimming the patch or the current gate—to independently assess the allocation of covert attention.

Although we focused our analysis, and this discussion, on differences in the magnitude of absolute shifts in the two conditions, it is worth considering that the *sign* is also reversed. That is, in the passive condition, we measure sensitivity to directly perceived shifts. Local motion to the left, causes a perceived shift to the left, and we measure sensitivity to leftwards deviation. In the active case, however, leftwards drift causes a compensatory adjustment of the patch to the right, and we measure deviation to the right. We thus have a nulling task in the active condition and direct judgment in the passive case. It is possible that fundamental differences in the nature of these tasks explains the change in magnitude. As already noted, the precise pattern of shifts has been shown to be very sensitive to the nature of the probe task (Kerzel and Gegenfurtner, 2005). One way to test this would be to design a passive task where left/right decisions at each subsequent gate adjust the position of the patch until it is perceived as centered. That is, replace the current method of constant stimuli with a “passive” nulling task.

On a related point, it is possible that the global motion involved in the nulling action and the local motion of the patch drift interact to affect perceived speed, either local or global. For example, the corrective action in the opposite direction to the local drift could cause an apparent increase in drift speed due to induced motion (Dunker, 1929; Bridgeman et al., 1981; Pas and Kappers, 2002). It is well known that the current illusory displacement scales with physical increases in local drift (De Valois and De Valois, 1991; Bressler and Whitney, 2006; Chung et al., 2007). Indeed, in our previous paper, we demonstrated similar physical speed scaling in the context of active control (Caniard et al., 2011). It would clearly be interesting to explore whether apparent increases in speed due to induced motion could have a similar effect, and thus play a role in the observed active-passive differences.

### Alternative explanations

So far, we have focused on aspects of our design that may have indirectly led to differences between the two conditions. In the remainder of this discussion, we will briefly consider a number of ways in which the magnitude of the illusion may have been influenced more directly. The suggested mechanisms/perspectives are not intended to be mutually exclusive and we freely acknowledge that at this stage we can only speculate as to the cause of apparent amplification. We raise the issues here purely as a possible guide to future research in this area.

First, as noted earlier in this discussion, it is clear that visual guidance of action must involve additional components relative to passive viewing, regardless of whether such components belong to one or two systems (Milner and Goodale, 1995; Glasser and Tadin, 2014). If any of these additional components are also susceptible to the illusion, then the current findings could result either from simple error summation or some form of interaction that amplifies the typically measured passive shift. To take a concrete example, the visuomotor control aspects of the active task must engage the cerebellum to a larger extent than the passive task (Brindley, 1964; Marr, 1969). In particular, the cerebellum is thought to influence fine motor control by helping to predict the sensory consequences of action (Ito, 1970; Kawato and Gomi, 1992; see Wolpert et al., 1998; Bastian, 2006 for overviews). If such predictive coding were also susceptible to the current illusion, this might contribute to the observed increase in overall shift. More generally, attempting to define the component stages in both passive and active tasks, possible via a physiologically plausible model (e.g., Giese and Poggio, 2003), and/or exploring clinical populations with relevant deficits (e.g., Synofzik et al., 2008) would seem to be productive avenues for future research.

Second, a common theme in many accounts of MIPS is the idea that illusions arise due to inherent delays in neural processing. As De Valois and De Valois (1991) noted, any amount of processing delay within the visual system effectively means that what we see “is not the world as it is now, but as it was in the near past” (p. 1625). Many illusions may thus arise as side effects of attempts to compensate for such delays. Some authors have proposed that delays are overcome by actively extrapolating the position of moving objects (e.g., Nijhawan, 1994), others that localization takes into account movement beyond the point in time at which an instantaneous estimate of position is triggered, so called motion biasing accounts (e.g., Eagleman and Sejnowski, 2007).

Recently, in the context of illusions, Marinovic et al. (2012) reemphasized that when *interacting* with moving objects, the goal is not just to determine where an object is now, but rather where it will be by the time a relevant action can be executed (e.g., Tresilian, 2005). Action plans thus need to account for both perceptual and motor delays. Marinovic et al. (2012) suggest such forward planning in the motor system could account for previous reports of action being more affected than perception in the context of MIPS (e.g., Yamagishi et al., 2001; Kerzel, 2003; Ashida, 2004). Although the authors were specifically referring to ballistic action to unseen, future locations, the general argument may also apply to the current task. That is, the large shift in the direction opposite to drift reported in our active task, may result not only from misestimating the instantaneous position of the patch, but by additional compensations for the assumed change of position during action execution. Marinovic et al. (2012) designed a task that was directly “interceptive”—moving a virtual paddle to contact a ball—which showed no errors, relative to a passive task in which the position of a bounce had to be estimated. Even though our current guidance task has an interceptive component—participants were required to “hit” the center of each gate—it is possible that other game-like tasks could be designed to specifically modualte the motor-prediction components of the illusion.

The third issue relates to the frame of reference used to code position in our two conditions. In very general terms, the position of an object can be coded either in retinotopic or spatiotopic coordinates. Recent evidence suggests that some motion illusions rely on the former framework, and some on the latter (Turi and Burr, 2012). Of most relevance here, however, are findings that the involvement of an active body-part can shift coding for a specific illusion away from a retinotopic frame of reference towards a body-centered one (Matsumiya and Shioiri, 2014). The standard visual motion aftereffect (MAE) is an illusion that follows adaptation to a moving pattern and normally requires retinal overlap between adaptor and test (Anstis et al., 1998; Turi and Burr, 2012). Matsumiya and Shioiri (2014) were able to demonstrate the illusion without retinal overlap when a participant’s active hand was a clearly visible reference point. Although hands were visible in both of our conditions, the actions involved in tilting the device may have placed more emphasis on body-centric coding. Shifts may thus be larger in the active condition either due to inherent differences in the precision of spatial coding in different frameworks or because our active task required the body parts to move, leading to re-calibration, which may have made localization more difficult/less precise.

Fourth and finally, it may be useful to consider behavior in our active task in terms of a simple, reactive vision system. Such systems are known to efficiently guide the behavior of many species. For example, flight control in insects has been well-documented (e.g., Götz, 1975; Reichardt and Poggio, 1975; Bülthoff, 1982; Hengstenberg et al., 1986), modeled (e.g., Cliff, 1992; Mura and Franceschini, 1994; Weber et al., 1997) and implemented as artificial systems (e.g., Huber et al., 1999; Neumann and Bülthoff, 2002). Braitenberg (1984) eloquently demonstrated how combinations of simple reactive mechanisms can interact with an environment to give rise to seemingly highly complex behavior and Bülthoff and Götz (1979) have specifically shown how such systems are susceptible to motion illusions. Within this framework, the closed-loop control structure of the current active task requires the display to be continuously sampled so that instantaneous assessments of patch position can be fed back and compared to the gate midline, the goal state. Deviations from this state must be detected and online adjustments made to the control parameters. Could aspects of this control structure—the rate at which position estimates are sampled, the nature of the online comparisons, the delay in parameter adjustment—lead to an amplification of shifts relative to the passive task? Behavioral experiments that limit the possible rate of sampling and/or perturb the control parameters, as well as virtual systems that simulate positional uncertainty are two example directions for future research that may help to shed light on the current findings.

## Conclusions

In the current paper we have introduced a new approach to studying a well-known visual illusion. The closed-loop control structure of our mobile app task provides a new technique for exploring the relationship between perception and action in the context of dynamic visual illusions, one that emphasizes the demands of active vision. By making the app freely available to other groups, we hope to provide a flexible framework within which other researchers can easily replicate and extend the current findings. In this initial study, our most compelling finding was the highly consistent amplification of the illusion when participants were actively controlling the target relative to when they were passively viewing. Although we have no definitive explanation for this pattern of results, we have outlined a number of research directions that can form the basis for future studies.

## Conflict of interest statement

The authors declare that the research was conducted in the absence of any commercial or financial relationships that could be construed as a potential conflict of interest.
